# Rac Inhibition Reverses the Phenotype of Fibrotic Fibroblasts

**DOI:** 10.1371/journal.pone.0007438

**Published:** 2009-10-13

**Authors:** Xu Shi-wen, Shangxi Liu, Mark Eastwood, Sonali Sonnylal, Christopher P. Denton, David J. Abraham, Andrew Leask

**Affiliations:** 1 Centre for Rheumatology, Department of Medicine, University College London (Royal Free Campus), London, United Kingdom; 2 The Canadian Institute of Health Research Group in Skeletal Development and Remodeling, Division of Oral Biology and Department of Physiology and Pharmacology, Schulich School of Medicine and Dentistry, University of Western Ontario, London, Ontario, Canada; 3 School of Biosciences, University of Westminster, London, United Kingdom; 4 University of Texas, M. D. Anderson Cancer Center, Houston, Texas, United States of America; Johns Hopkins School of Medicine, United States of America

## Abstract

**Background:**

Fibrosis, the excessive deposition of scar tissue by fibroblasts, is one of the largest groups of diseases for which there is no therapy. Fibroblasts from lesional areas of scleroderma patients possess elevated abilities to contract matrix and produce α−smooth muscle actin (α-SMA), type I collagen and CCN2 (connective tissue growth factor, CTGF). The basis for this phenomenon is poorly understood, and is a necessary prerequisite for developing novel, rational anti-fibrotic strategies.

**Methods and Findings:**

Compared to healthy skin fibroblasts, dermal fibroblasts cultured from lesional areas of scleroderma (SSc) patients possess elevated Rac activity. NSC23766, a Rac inhibitor, suppressed the persistent fibrotic phenotype of lesional SSc fibroblasts. NSC23766 caused a decrease in migration on and contraction of matrix, and α−SMA, type I collagen and CCN2 mRNA and protein expression. SSc fibroblasts possessed elevated Akt phosphorylation, which was also blocked by NSC23766. Overexpression of rac1 in normal fibroblasts induced matrix contraction and α−SMA, type I collagen and CCN2 mRNA and protein expression. Rac1 activity was blocked by PI3kinase/Akt inhibition. Basal fibroblast activity was not affected by NSC23766.

**Conclusion:**

Rac inhibition may be considered as a novel treatment for the fibrosis observed in SSc.

## Introduction

Fibrosis is characterized by excessive deposition of scar tissue. Fibrosis is one of the largest groups of diseases for which there is no therapy. It has been estimated that nearly 45% of all deaths in the developed world are caused fibrotic conditions which include: cardiovascular disease, pulmonary fibrosis, diabetic nephropathy and liver cirrhosis [1]. An example of a chronic fibrotic disease is systemic sclerosis (SSc, scleroderma) which, in its diffuse form, can affect the skin and internal organs such as the lung and kidney, resulting in significant morbidity [2]. Although the fundamental cause for fibrotic conditions including SSc is unclear, a common theme of these diseases is the abnormal persistence of a particular specialized form of fibroblast, termed the myofibroblast [3]–[5]. The myofibroblast expresses a highly contractile form of actin, α-smooth muscle actin (α−SMA) which is connected to the extracellular matrix (ECM) through specialized cell surface structured called focal adhesions (FAs) [3], [4]. Thus, the myofibroblasts can exert mechanical tension on the ECM [6]–[8]. The myofibroblast is considered to be responsible for the excessive production, adhesion and contraction of ECM characterizing fibrotic lesions [6], [7].

Several different cytokines and extracellular proteins have been identified that contribute to myofibroblast formation [e.g., transforming growth factor β (TGFβ) endothelin-1, platelet derived growth factor, Angiotensin, connective tissue growth factor(CTGF, CCN2)] [9]–[13]. Considering SSc, studies using specific inhibitors of individual cytokines inhibitors have been found to be partially effective at alleviating the persistent fibrotic phenotype of fibrotic fibroblasts; moreover, individual cytokines appear to be responsible for complementary, overlapping features of SSc fibroblasts [14]–[17]. Thus, additional strategies may be warranted.

It is now appreciated that FA proteins can serve as a point of convergence for signals emanating from stimulated growth factor receptors [8], [18]. As a specific example, the FA protein paxillin can bind focal adhesion kinase (FAK) [19], and is responsible for the recruitment of Rac1, a member of the Rho family of small GTPases [20], [21], to FAs [22]. Rac1 is required for fibroblast migration both in vitro and in vivo [23]–[26]. We have shown that mice possessing a fibroblast-specific deletion of Rac1 exhibit impaired myofibroblast formation and function, associated with delayed cutaneous tissue repair and resistance to bleomycin-induced fibrosis [26], [27].

The results described above strongly indicate that targeting myofibroblast action by pharmacological inhibition of Rac may represent a novel approach to inhibiting fibrosis. Dermal fibroblasts cultured from lesional areas of SSc patients retain their fibrotic phenotype for several passages in culture [5], [28], [29]. In this report, we test the hypothesis that inhibition of Rac may reverse the persistent fibrotic phenotype of fibroblasts cultured from lesional areas of scleroderma patients. Our data provide new and valuable insights into the fundamental basis of the fibrotic phenotype of SSc fibroblasts and suggest a possible new course of therapy for SSc.

## Methods

### Cell culture, transfection and Western analysis

Dermal fibroblasts were isolated from by explant culture of 4 mm punch biopsies from the forearm of healthy individuals and those with diffuse cutaneous scleroderma (6 each) in DMEM, 10% fetal bovine serum (Invitrogen) as previously described [4], [28]. Donors were age-, site- and sex-matched. Experimental protocols were approved by the Ethics Committee of the Royal Free Hospital where all participants were recruited, under informed written consent, and human experimentation was conducted. Cells (∼80% confluence) were serum-starved overnight, lysed in 2% SDS, and proteins quantified (Pierce) and subjected to Western blot analysis as previously described [5], [21]. Antibodies used were: phospho-Akt, Akt (Cell Signaling), type I collagen (Biodesign), GAPDH (Sigma) and secondary antibodies were from Abcam. Alternatively, RNA was harvested using Trizol as described by the manufacturer (Invitrogen). When indicated, cells were transfected (Fugene) with empty expression vector or expression vector encoding active rac1 (courtesy Alan Hall, University College London) in a ratio of 2 µg DNA:3 µl Fugene as described by the manufacturer and previously [21]. Mouse fibroblasts deleted (K/K) or not (C/C) for rac1 were previously generated and were cultured as previously described [27]. When indicated, cells were treated with DMSO (vehicle) or the rac inhibitor NSC23766 (50 µM in DMSO; Calbiochem), a rac-specific small molecule which specifically binds to rac proteins and prevents their activation at this concentration in fibroblasts [30], [31].

### Real time polymerase chain reaction (RT-PCR)

Dermal fibroblasts were serum-starved for 18 h and treated in the presence or absence of 10 µM bosentan for 24 h. Total RNA was isolated using Trizol (Invitrogen), and the integrity of the RNA was verified by gel electrophoresis. Total RNA (10 µg) was reverse transcribed in a 20-µl reaction volume containing an oligonucleotide (dT_18_) and random decamers (dN_10_) using M-MLV reverse transcriptase (Promega) for 1 h at 37°C. Primers for SMA, Col1A2, CTGF and Vinculin were as previously described [32], [33]. The cDNA was diluted to 100 µl with diethylpyrocarbonate-treated water, and the target was measured by real-time PCR FastStart DNA Master SYBR Green (Roche Applied Science) according to the manufacturer's instructions. Triplicate samples were run, transcripts were measured in picograms, and expression values were standardized to values obtained with control 28S RNA. Alternatively, total RNA (25 ng) was reverse transcribed and amplified using TaqMan Assays on Demand (Applied Biosystems) in a 15-µl reaction volume containing two unlabeled primers and 6-carboxyfluoroscein labeled TaqMan MGB probe. Samples were combined with TaqMan one-step mastermix (Applied Biosystems). Amplified sequences were detected using the ABI Prism 7900 HT sequence detector (Perkin-Elmer-Cetus, Vaudreuil, QC) according to the manufacturer's instructions. Triplicate samples were run, and expression values were standardized to values obtained with control 28 S RNA primers.

### Collagen gel contraction

Experiments were performed essentially as described previously [34]. Briefly, 24-well tissue culture plates were pre-coated with BSA. Cells were used at passage 3. Trypsinized fibroblasts were suspended in MCDB medium (Sigma) and mixed with collagen solution (one part of 0.2 M N-2-hydroxyethylpiperazine-N′-2-ethanesulfonic acid [HEPES], pH 8.0; four parts collagen [Vitrogen-100, 3 mg/ml] and five parts of MCDB X 2) yielding a final concentration of 80,000 cells per ml and 1.2 mg/ml collagen. Collagen/cell suspension (1 ml) was added to each well. After polymerization, gels were detached from wells by adding 1 ml of MCDB medium. Contraction of the gel was quantified by measuring changes in gel weight, which is routinely used as a measurement of gel contraction [34]; the weight arises due to the media absorbed into the collagen gel. In parallel, gel contraction was measured by calculating the diameter of the contracted gel; results were identical to those obtained when gel weight was measured.

### Fibroblast populated collagen lattice (FPCL) contraction assay

Measurement of contractile forces generated in a tethered, floating fibroblast-populated collagen lattice (FPCL) was performed as described [34], [35]. Cells (1×10^6^ cells/ml) were seeded into a collagen gel (First Link), floated in DMEM, 2% FBS, and forces generated across the collagen lattice were measured over a 24 hour period. Graphical readings are produced every 15 seconds providing continuous measurements of generated forces (Dynes: 1×10^−5^ N) [13], [14], which are logged into a personal computer.

### Migration assays

Cultured fibroblasts were cultured in 12-well plates. When cells were confluent, medium was removed, cells were rinsed with serum-free medium + 0.1% BSA and cultured for an additional 24 h in serum-free medium + 0.1% BSA. A uniform scratch was them made across the plate with a blue pipette tip. Cells were washed two times in PBS, and cultured in serum-free medium in the presence of mitomycin C (10 µg/ml, Sigma).

### Rac activity assay

A rac-GTP pulldown assay was used, essentially as previously described (36; Upstate Biotechnology; Lake Placid, NY). Briefly, fibroblasts were grown in 60 mm dishes and protein was harvested by lysis in buffer containing NP-40. Agarose beads to which a PAK-GSH fusion protein was conjugated was added and active Rac (Rac-GTP), which binds PAK-GSH, was recovered through repeated centrifugation and washing of the agarose beads. Bound Rac was identified by boiling beads in Laemmli buffer, and subjecting the resultant extracts to SDS-PAGE and Western blot analysis using anti-Rac antibody. When indicated, cell lysates were stimulated with GTPγS (100 µmol/L) as a control to establish maximal Rac activation.

## Results

### The Rac inhibitor NSC23766 reduces the overexpression of pro-fibrotic genes in SSc fibroblasts

**Figure 1:**
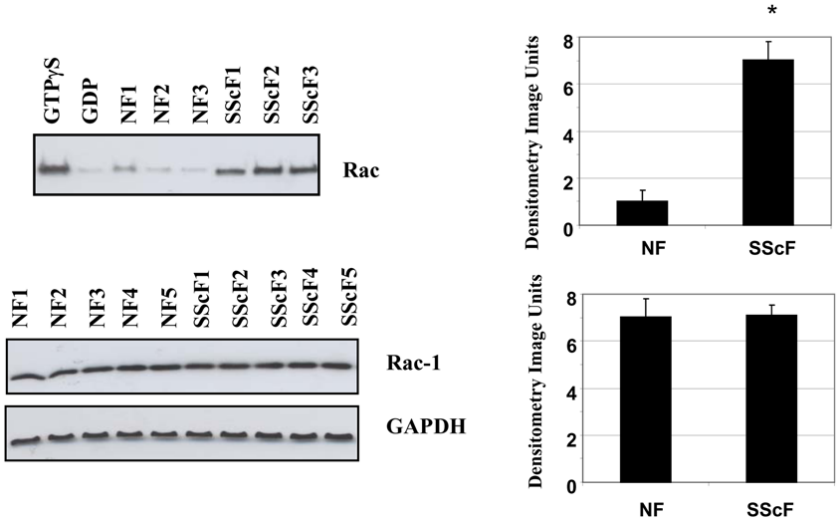
Rac activity is elevated in dermal fibroblasts cultured from scars of SSc patients. Cells were loaded with or without GTPγS (positive control, to assess maximal Rac activation) or GDP (as a negative control). Rac-GTP was immunoprecipitated from cell lysates. The precipitated Rac-GTP was detected by immunoblot analysis using anti-Rac. In parallel, whole cell lysates were subjected to SDS/PAGE and Western blot analysis with an anti-rac antibody. Quantitative densitometry data is indicated on the right. Lanes are: GTPγS, GDP, cells from three normal individuals (N1, N2, N3) and three individuals with SSc (SScF1, SScF2, SScF3). Experiments were performed on cells derived from 6 normal individuals and 6 SSc patients. Quantitative densitometry data is indicated on the right (* = p<0.05 relative to wild-type control).

**Figure 2:**
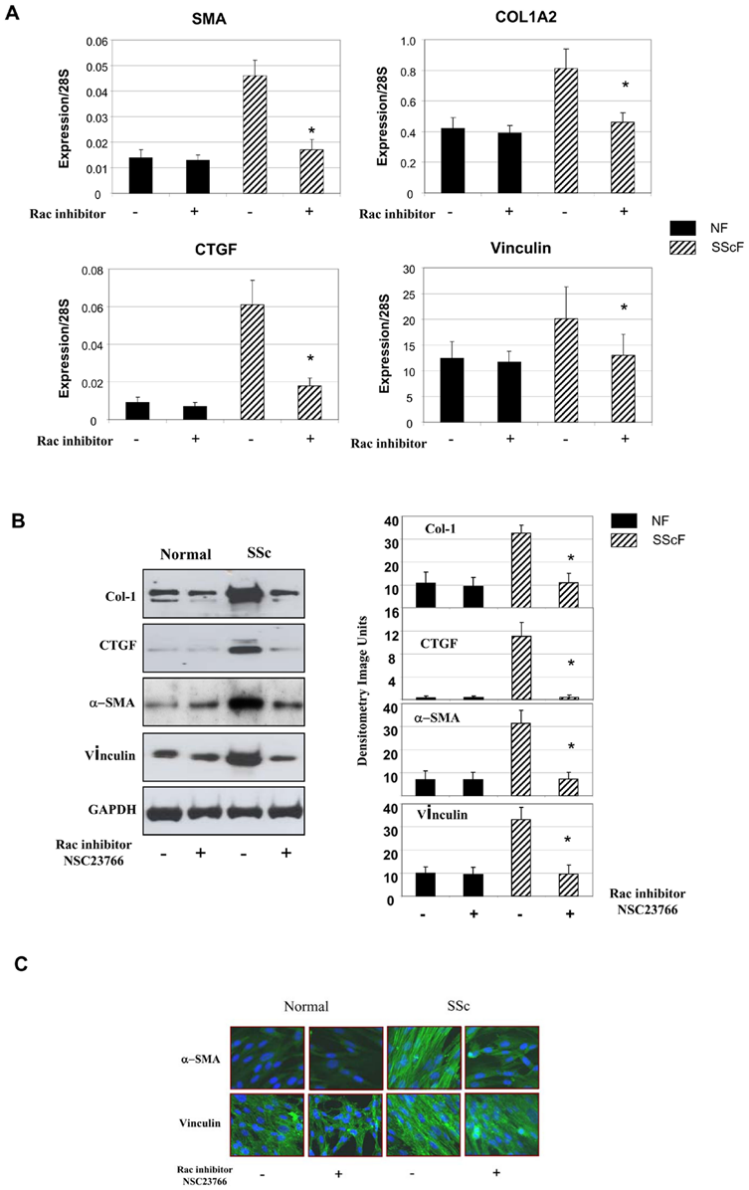
Rac inhibition suppresses the pro-fibrotic phenotype of lesional SSc fibroblasts. mRNA and protein analysis. Fibroblasts were from normal individuals (NF) and individuals with SSc (SScF) were assessed in the presence or absence of the rac-specific inhibitor (NSC23766, 24 hour treatment 50 µM). (A) Real-time PCR analysis. Messenger RNA was harvested from cells and subjected to real-time PCR analysis to detect the mRNAs indicated. Fibroblasts from 6 individuals were analyzed. Data represent averages and standard deviation. As a control, 28S RNA was amplified. Average of three replicates from three separate individuals, adjusted for 28S RNA expression values, are shown (+/− SD * = *p*<0.05 significant inhibition by NSC23766 compared to untreated controls, Student's paired t test). (B) Western blot analysis. Proteins were harvested and subjected to Western blot analysis with antibodies directed against the proteins indicated. Densitometry is on the right (+/− SD, * = *p*<0.05 significant inhibition by NSC23766 compared to untreated controls, Student's paired t test) (C) Immunofluorescence analysis. Cells were fixed and stained with anti-vinculin antibody to detect focal adhesion and rhodamine phalloidin to detect actin and α-SMA.

To extend our previous data showing that rac1 was required for bleomycin-induced skin fibrosis [26], we first used a standard, commercially available rac activity assay to show that rac activity was elevated in SSc dermal fibroblasts compared to control fibroblasts (Figure 1). Conversely, rac expression was not altered in SSc fibroblasts (Figure 1). To begin to assess whether heightened rac activity contributed to the enhanced fibrotic phenotype of lesional SSc fibroblasts, we used real-time PCR analysis to show, as expected, that α-SMA, type I collagen (COL1A2), vinculin and CTGF mRNAs were elevated in SSc dermal fibroblasts (SScF) compared to control fibroblasts (NF) (Figure 2A). Cells incubated for 24 hours with the rac- specific inhibitor NSC23766 (50 µM) showed reduced α-SMA, type I collagen (COL1A2), vinculin and CTGF mRNA expression (Figure 2A). NSC23766 did not appreciably affect mRNA expression in normal fibroblasts (Figure 2A). Similar results were obtained when protein expression was assessed using Western blot analysis; NSC23766 reduced α−SMA, type I collagen, vinculin and CTGF protein expression in SSc fibroblasts (Figure 2B). Consistent with these data, NSC23766 was able to reduce the appearance of α-SMA-containing stress fibers in SSc fibroblasts (Figure 2C). These results are consistent with the notion that enhanced rac activity by lesional SSc fibroblasts contributes to the fibrotic phenotype of lesional SSc fibroblasts.

### The Rac inhibitor NSC23766 reduces the enhanced ability of lesional SSc fibroblasts to contract a collagen gel matrix

**Figure 3:**
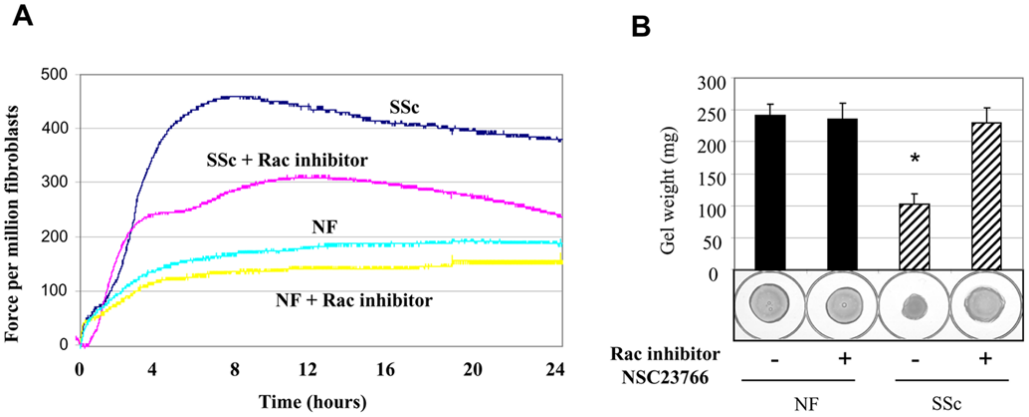
Rac inhibition suppresses the pro-fibrotic phenotype of lesional SSc fibroblasts. Gel contraction analysis. Rac inhibitor 50 µM NSC23766 (24 hour treatment) reduced the ability of lesional SSc fibroblasts to contract a collagen gel matrix: (A) FPCL analysis. The effect of loss of rac inhibition on the ability of fibroblasts to exert contractiles force in a fixed, tethered floating collagen gel lattice was investigated using a Culture Force Monitor. Forces generated by fibroblasts were measured over 24 hours; a representative trace is shown (N = 3). (B) Floating gel analysis. The effect of rac inhibition on ECM contraction over 24 hours generated by fibroblasts embedded in a floating collagen gel matrix was evaluated. Contraction was assessed photographically and by measuring gel weight iameter of contracted gels (n = 6; Average +/− standard deviation is indicated; * = p<0.05, statistically different from control untreated normal fibroblasts, ANOVA). Note that NSC23766 suppressed the enhanced ECM contraction by lesional SSc fibroblasts.

To provide a functional context for our experiments, we then assessed whether rac inhibition could alleviate the elevated ability of lesional SSc fibroblasts to contract a collagen gel matrix. First, we showed, as expected, that lesional SSc fibroblasts, relative to control dermal fibroblasts, showed an enhanced ability to contract a floating collagen gel matrix (Figure 3A) and to generate contractile forces across a fibroblast populated collagen gel lattice which was fixed at one end (Figure 3B). NSC23766 (24 hours; 50 µM) inhibited the enhanced ability of SSc fibroblasts to contract a floating collagen gel, as well as exert contractile forces across a collagen gel matrix (Figure 3A,B). These results provide further support to the hypothesis that enhanced rac activity by lesional SSc fibroblasts contributes to the fibrotic phenotype of lesional SSc fibroblasts.

### The Rac inhibitor NSC23766 reduces the enhanced ability of lesional SSc fibroblasts to migrate

**Figure 4:**
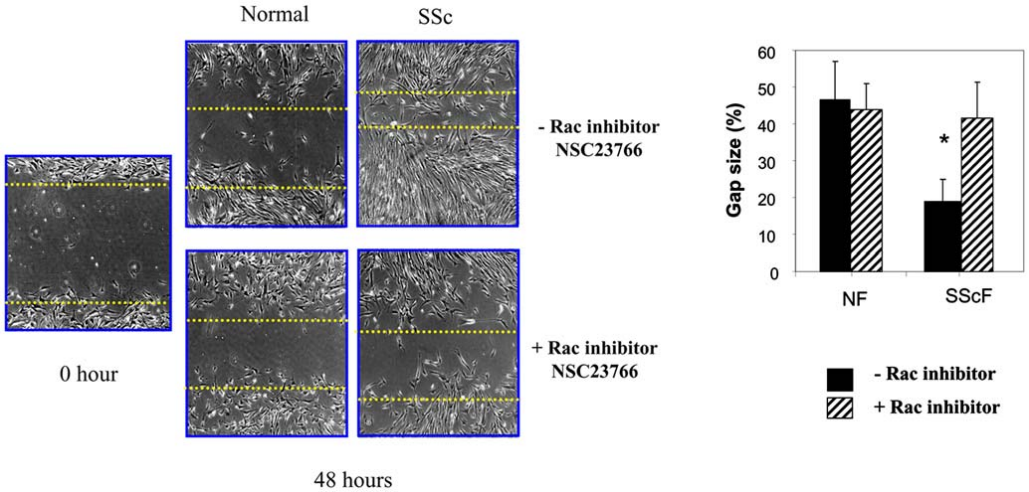
Rac inhibition reduces the enhanced migration of lesional SSc fibroblasts. Fibroblasts were subjected to a scratch wound assay in the presence or absence of 50 µM NSC23766. Cells were cultured on fibronectin until reaching confluence. A uniform linear scrape wound was made across the cell layer. Three independent experiments were performed Gap size expressed as a percentage of the original wound is shown (average +/− standard deviation) (* = p<0.05 statistically different relative to untreated healthy controls).

To assess whether enhanced rac activity may contribute to this phenomenon, we cultured dermal fibroblasts isolated from SSc patients and healthy individuals until confluence. A uniform scratch wound was created across the cell layer, and migration monitored over 48 hours in the presence or absence of NSC23766. SSc cells (SScF) migrated faster than control fibroblasts (NF) (Figure 4). However, incubation of cells with NSC23766 suppressed the enhanced migratory ability of SSc fibroblasts to that of controls (Figure 4). Again, rac inhibition had no appreciable effect on the migration of normal fibroblasts (Figure 4). Collectively, these data lead additional credence to the notion that the fibrotic phenotype of lesional SSc fibroblasts is due to enhanced rac activity.

### Rac1 directly promotes a fibrogenic phenotype in fibroblasts via PI3kinase/Akt

**Figure 5:**
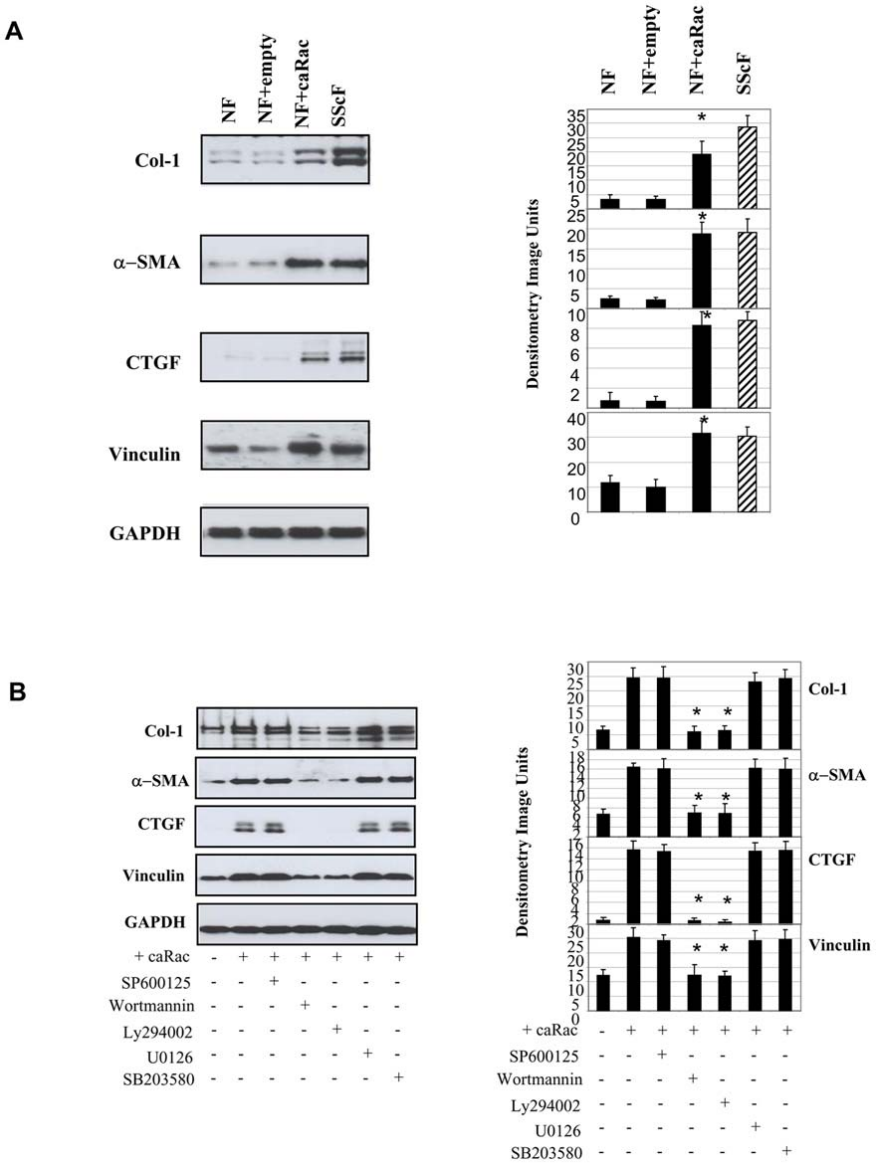
Overexpression of rac in normal fibroblasts results in a profibrotic phenotype in a PI3kinase/Akt-dependent fashion: protein analysis. (A) Transfection of constitutively active rac (ca rac), compared to empty expression vector (empty), increases profibrotic protein expression. Twenty four hours post-transfection, protein was harvested and subjected to Western blot analysis was conducted with antibodies detecting the proteins indicated. Average +/− standard deviation is shown (N = 3, * = p<0.05). (B) The effect of rac overexpression is reduced by PI3 kinase inhibition. Cells were transfected with empty expression vector or constitutively active rac (ca rac) in the presence or absence of 100 nM wortmannin, 10 µM Ly294002, 10 µM U0126 and 10 µM SP600125. Cells were then processed for Western blot analysis as described in (A).

To assess whether activation of rac was sufficient to generate a fibrogenic phenotype, we transfected into normal fibroblasts (NF) either an empty expression vector or an expression vector encoding constitutively active rac1 (NF+empty versus NF+caRac). We then performed western blot analysis on protein extracts prepared from cells 24 hours post-transfection. In parallel, western blot analysis was performed on extracts prepared from untransfected SSc fibroblasts (SScF). We found that, compared to empty expression vector, transfection of an expression vector encoding constitutively active rac1 into normal fibroblasts resulted in increased production of type I collagen, α−SMA, CTGF and vinculin protein (Figure 5A).

**Figure 6:**
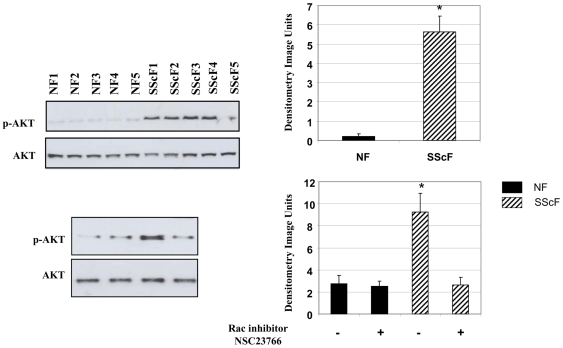
Akt phosphorylation is elevated in dermal fibroblasts and is reduced by rac inhibition. Whole cell lysates were subjected to SDS/PAGE and Western blot analysis with an anti-phospho Akt and anti-Akt antibodies in the absence (TOP PANEL) or presence or absence (BOTTOM PANEL) of 50 µM NSC23766. Quantitative densitometry data is indicated on the right. Experiments were performed on cells derived from 6 normal individuals and 6 SSc patients. Quantitative densitometry data is indicated on the right (* = p<0.05 relative to wild-type control).

We then wished to examine the molecular mechanism underlying the ability of rac1 to promote a fibrogenic phenotype in fibroblasts. To begin to address this issue, we tested the hypothesis that rac might act through the activation of PI3kinase/Akt. We first used Western blot analysis to assess whether SSc fibroblasts displayed elevated phosphorylation of Akt. Cells were incubated overnight in serum free media in the presence or absence of NSC23766. Western blot analysis of the resultant protein whole cell extracts revealed that SSc fibroblasts possessed elevated phosphorylation of Akt, which was suppressed by the presence of NSC23766 (Figure 6A, B). Thus SSc fibroblasts displayed elevated Akt activation in a rac-dependent fashion.

We then investigated if the ability of overexpressed active rac1 to activate profibrotic protein expression in fibroblasts was blocked by PI3kinase/Akt inhibition. To perform this expreriment, we repeated our transfection experiments, this time in the presence or absence of the JNK inhibitor SP600125, the p38 inhibitor SB203580, the MEK inhibitor U0126 or the PI3 kinase inhibitors wortmannin and Ly294002. We showed that, of these inhibitors, only wortmannin and Ly294002 blocked the ability of activated rac1 to increase profibrotic protein expression indicating that rac1 acted via PI3kinase (Figure 5B).

**Figure 7:**
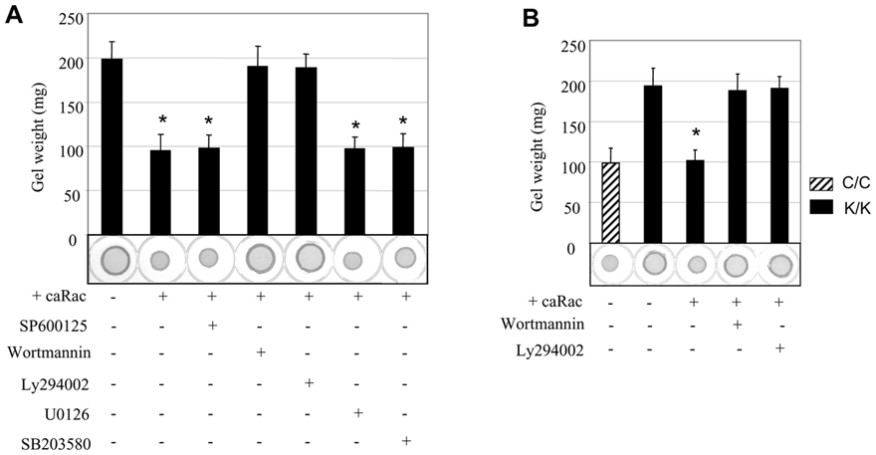
Overexpression of rac in normal fibroblasts results in a profibrotic phenotype phenotype in a PI3kinase/Akt-dependent fashion: collagen gel contraction analysis. (A) Transfection of constitutively active rac (ca rac), compared to empty expression vector (empty), increases ECM contraction by normal fibroblasts. Eighteen hours post-transfection, cells were subjected to the floating collagen gel model of ECM contraction in the presence or absence of 100 nM wortmannin, 10 µM Ly294002, 10 µM U0126 or 10 µM SP600125. Average +/− standard deviation is shown (N = 3, * = p<0.05). (B) Rac overexpression rescues the reduced ECM contraction in rac knockout fibroblasts (K/K) versus control fibroblasts (C/C). Cells were transfected with empty expression vector or constitutively active rac (ca rac) in the presence or absence of wortmannin or Ly294002. (*, p<0.05, Student's t test).

To extend these data, we showed that overexpression of rac1 could increase the ability of normal fibroblasts to contract a floating collagen gel matrix, and that this ability was blocked by wortmannin and Ly294002 but not JNK inhibitor SP600125, the p38 inhibitor SB203580 or the MEK inhibitor U0126 (Figure 7A). Finally, previously we had shown that mouse skin fibroblasts deleted for rac1 were less able to contract a floating collagen gel matrix relative to control fibroblasts [27]. We confirmed these data (K/K versus C/C; Figure 7B), and showed that overexpression of rac1 rescued this phenotype, in a fashion which was sensitive to wortmannin and Ly294002 (Figure 7B). Collectively, these data suggest that activated rac can directly promote a fibrogenic phenotype through PI3kinase/Akt and that targeting rac may be a viable therapy for fibrosis, including that observed in SSc.

## Discussion

Fibrotic diseases are characterized by the persistence of myofibroblasts within lesions [3]–[5]. Altered activities of Rho GTPases, such as Rac, have been proposed to contribute to the pathology of diseases such as cancer, and, as such, have been proposed as potential drug targets [35]. Previously, we have shown, using a conditionally deleted Rac1 allele, that Rac1 is required for proper cutaneous tissue repair kinetics and for bleomycin to promote skin fibrosis [26], [27]. In these studies, we showed that Rac1 was required for myofibroblast differentiation and activity. In this report, we extend these studies and show that a rac inhibitor reverses the phenotype of lesional fibroblasts isolated from the skin of scleroderma patients. These results suggest that anti-Rac strategies may be useful anti-fibrotic treatments. Future efforts examining the potential utility of rac inhibition as a strategy in SSc will require an assessment of the specificity of rac inhibition in selectively blocking fibrogenic responses in vitro and an in vivo prior to the translation of this knowledge into humans. In this light, it is important to note that rac is known to mediate a variety of functions including cytoskeleton organization, transcription, and cell proliferation in immune, vascular and epithelial systems [38]. Nonetheless, these data support the general notion that targeting proteins located within cells that are required for myofibroblast action may be a viable novel anti-fibrotic approach. This novel approach is required, as strategies blocking extracellular factors promoting myofibroblast differentiation and fibroblast activation have not been shown to be effective thus far [39].

Akt (also called protein kinase B, PKB), a serine/threonine kinase consisting of three members termed PKB*α* (Akt1), PKBβ (Akt2), and PKBγ (Akt3), is activated in cells in response to a variety of stimuli including hormones, growth factors, and ECM [40], [41]. Akt activation occurs in response to PI3-kinase activation. PI3-kinases contribute to a variety of cellular processes including cell survival, migration cytoskeletal remodeling and metabolic control [42], [43]. Our data showing that Akt was elevated in SSc cells are consistent with previously published observations from a different group [44]. However, that rac activity is elevated in SSc cells and that this is required for enhanced Akt activity in SSc fibroblasts has not been shown. Moreover, in this current report we also provided data that rac1-dependent activity was blocked by PI3kinase/Akt inhibition. Our current results are consistent with previous observations that endothelin-1 promotes ECM contraction in fibroblasts via rac and PI3kinase/Akt [34].

In summary, our studies examining the involvement in Rac1 in dermal function may have profound implications for both homeostatic and pathological processes by contributing to our understanding of basic mechanisms regarding wound healing. As a consequence, our results may have future therapeutic implications for the treatment of non-healing or chronic skin wounds, and of fibroproliferative disease.
